# Substrate strain tunes operando geometric distortion and oxygen reduction activity of CuN_2_C_2_ single-atom sites

**DOI:** 10.1038/s41467-021-26747-1

**Published:** 2021-11-03

**Authors:** Guokang Han, Xue Zhang, Wei Liu, Qinghua Zhang, Zhiqiang Wang, Jun Cheng, Tao Yao, Lin Gu, Chunyu Du, Yunzhi Gao, Geping Yin

**Affiliations:** 1 MIIT Key Laboratory of Critical Materials Technology for New Energy Conversion and Storage, School of Chemistry and Chemical Engineering, Harbin Institute of Technology, Harbin, PR China; 2 State Key Laboratory of Physical Chemistry of Solid Surfaces, Collaborative Innovation Center of Chemistry for Energy Materials, National Engineering Laboratory for Green Chemical Productions of Alcohols, Ethers and Esters, College of Chemistry and Chemical Engineering, Xiamen University, Xiamen, PR China; 3 Center for Materials and Interfaces, Shenzhen Institutes of Advanced Technology, Chinese Academy of Sciences, Shenzhen, PR China; 4 National Synchrotron Radiation Laboratory, University of Science and Technology of China, Hefei, PR China; 5 Beijing National Laboratory for Condensed Matter Physics, Institute of Physics, Chinese Academy of Sciences, Beijing, PR China; 6 Department of Chemistry, University of Western Ontario, London, ON Canada

**Keywords:** Nanoscale materials, Fuel cells, Electrocatalysis

## Abstract

Single-atom catalysts are becoming increasingly significant to numerous energy conversion reactions. However, their rational design and construction remain quite challenging due to the poorly understood structure–function relationship. Here we demonstrate the dynamic behavior of CuN_2_C_2_ site during operando oxygen reduction reaction, revealing a substrate-strain tuned geometry distortion of active sites and its correlation with the activity. Our best CuN_2_C_2_ site, on carbon nanotube with 8 nm diameter, delivers a sixfold activity promotion relative to graphene. Density functional theory and X-ray absorption spectroscopy reveal that reasonable substrate strain allows the optimized distortion, where Cu bonds strongly with the oxygen species while maintaining intimate coordination with C/N atoms. The optimized distortion facilitates the electron transfer from Cu to the adsorbed O, greatly boosting the oxygen reduction activity. This work uncovers the structure–function relationship of single-atom catalysts in terms of carbon substrate, and provides guidance to their future design and activity promotion.

## Abstract (web summary)

The rational design of single-atom catalysts is challenging. This work reveals a substrate-strain tuned geometry distortion of CuN_2_C_2_ single-atom site, which greatly boosts oxygen reduction activity by facilitating electron transfer to adsorbed O.

## Introduction

Single-atom catalysts (SACs) dispersed on conductive carbon substrates are granted rich redox and coordination chemistry of metal centers, and present unique built-in catalytic activity and selectivity in numerous energy conversion reactions such as oxygen reduction^1–3^, oxygen evolution^4–6^, and carbon dioxide reduction^7–9^. Accordingly, the single-atom catalysis has become the most active frontier in energy conversion catalysis in the past decade^10–13^. Despite tremendous efforts, the general try-and-error methodology of constructing single-atom sites lacks the efficiency in tuning the activity and selectivity of SACs. Comprehensively understanding the catalytic behavior and structure–function relationship of single-atom active sites is a prerequisite for the rational design and construction of highly efficient SACs. This understanding, however, remains a great challenge because of the complicated active site structures and the complex reaction conditions of SACs.

As the latest development of understanding the catalytic behavior of SACs, the dynamic evolution of active sites in operando processes has recently been probed using advanced characterization techniques, especially X-ray absorption spectroscopy (XAS)^14–16^. For instance, the Fe center in Fe–N–C SACs is found to undergo Fe^2+^/Fe^3+^ transition, and the ferrous state is the real active site during the oxygen reduction reaction (ORR)^17,18^. For the RuN_4_ SACs catalyzing the oxygen evolution reaction, the adsorption of extra O is observed to induce the in situ reconstruction of active site, and the formed O-RuN_4_ moiety highly enhances the activity^5^. These dynamic catalytic behaviors provide valuable information on the determination of active sites and the comprehension of reaction mechanism. However, the detailed structure–function relationship underneath diverse catalytic behaviors is still unclear. In particular, the correlation between the carbon substrate structure and the operando catalytic behavior has not yet been paid much attention, although previous studies have proven that carbon substrates have strong effects on the properties of SACs^19–22^.

**Fig. 1:**
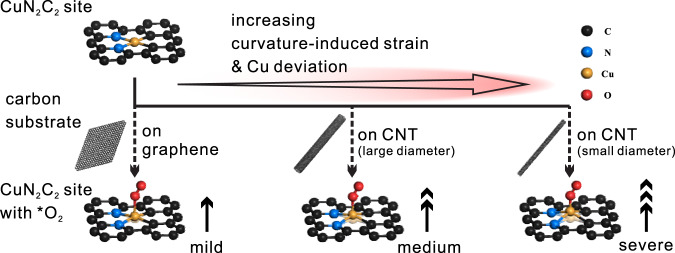
Illustration of structural distortion of CuN_2_C_2_ active site with adsorbed O_2_ on different *sp*^2^-hybridized carbon frameworks. The distortion becomes more severe as the curvature-induced strain increases in the substrate from graphene (left) to CNT with small diameter (right).

Graphene and carbon nanotube (CNT) have definite and uniform *sp*^2^-hybridized carbon framework structures, providing ideal model substrates to investigate the structure–function relationship of SACs. Compared with graphene, CNT possesses more *sp*^3^-like C atoms and strained C–C bonds due to the geometric bending^23–25^. The bended carbon substrates possibly lead to distinctive dynamic evolution of active sites and thus manipulate the catalytic processes of SACs^26,27^. In this work, we try to correlate the operando structure evolution of SACs to their catalytic properties, and demonstrate the substrate-induced activity enhancement of CuN_2_C_2_ SACs embedded within *sp*^2^-hybridized carbon frameworks. Both theoretical computation and operando XAS indicate that the CuN_2_C_2_ active site is geometrically distorted during the ORR as a response to the newly coordinated O-containing species, and the active site on the highly curved CNT substrates undergoes more severe distortion to release strain. Taking advantage of the strain of CNT substrate, we are able to manipulate the geometric distortion of CuN_2_C_2_ active site (Fig. 1). This distortion artfully balances the two contrary effects of strengthening the new Cu–O bonding and weakening the original Cu–N/Cu–C bonding to tune the electron transfer to the adsorbent. As a result, the CuN_2_C_2_ active site on the reasonable CNT substrate presents the optimized geometric distortion for achieving the most electrons transferred to the adsorbed O_2_ molecules, thus enhancing the ORR activity of up to sixfold. Our work reveals the strain-induced geometric distortion and the substrate structure–activity relationship of CuN_2_C_2_ active site, paving a new pathway to the future rational design and further activity promotion of SACs.

## Results

### Fabrication and ORR performance

The CuN_2_C_2_ single-atom active site on the *sp*^2^-hybridized carbon substrates was fabricated using a confined self-initiated dispersing protocol as described in our previous work^28^. These single-atom Cu moieties are in situ formed during the high temperature pyrolysis, and have a quasi-planar coordination geometry, corresponding to an embedded structure within the carbon framework^29^. Graphene and two representative sizes of CNT with the diameter of 8 and 4 nm were utilized as the substrates, and the corresponding SACs are denoted as Cu/G, Cu/CNT-8, and Cu/CNT-4, respectively.

**Fig. 2:**
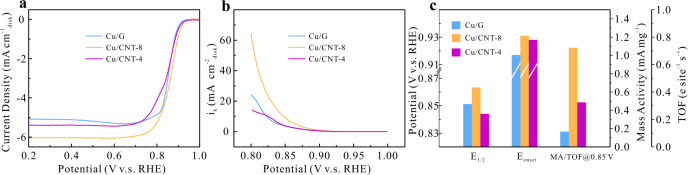
Oxygen reduction activity of Cu/G and two Cu/CNT samples. **a** ORR polarization curves, **b** kinetic current density curves, and **c** *E*_1/2_, *E*_onset_, MA, and TOF at 0.85 V.

The catalytic activity of these Cu-SACs toward ORR was evaluated in O_2_-saturated 0.1 M KOH solution in a standard three-electrode quartz cell with catalyst-coated glassy carbon (GC) rotating-disk electrode, Hg/HgO electrode and Pt foil as the working, reference and counter electrodes, respectively (see details in Methods). Among the three catalysts, Cu/CNT-8 presents the most positive ORR polarization curve (Fig. 2a). To quantitatively assess the ORR activity, kinetic current density (*i*_k_) was calculated based on the K–L plots^30^ (Supplementary Fig. 1) and is shown in Fig. 2b. In the whole kinetics control and mixed kinetics-diffusion control regions from ~0.92 to ~0.8 V, *i*_k_ of Cu/CNT-8 is apparently higher than that of the other two samples. Specifically, *i*_k_ at 0.85 V for Cu/CNT-8 reaches 9.24 mA cm^−2^, which is more than 2.5 times that for Cu/G (3.62 mA cm^−2^).

The onset potential (*E*_onset_) and half-wave potential (*E*_1/2_) are compared in Fig. 2c. *E*_onset_ (0.933 V) and *E*_1/2_ (0.863 V) of Cu/CNT-8 surpass Cu/G by 13 and 12 mV, respectively, confirming that Cu/CNT-8 is the most active towards the ORR and even comparable to some reported Fe- and Co-based SACs (Supplementary Table 1). Moreover, to normalize the ORR activity by the amount of Cu single atoms, mass activity (MA), and turnover frequency (TOF) were calculated based on the inductively coupled plasma optical emission spectroscopy (ICP-OES) results (Supplementary Table 2), and are further compared in Fig. 2c^31^. Cu/CNT-8 exhibits the highest MA of 1.08 A mg^−1^ and TOF of 0.72 e·s^−1^·site^−1^ at 0.85 V, which are more than 6 times those of Cu/G (0.17 A mg^−1^ and 0.11 e site^−1^ s^−1^, respectively), outperforming most of the Cu-based active moieties in SACs (Supplementary Table 1). Noteworthy is that a much lower ORR activity is observed for Cu/CNT-4 in terms of onset potential, half-wave potential, MA and TOF even though it also employs CNT as the substrate, and similar compromise on ORR performance can also be seen on larger-sized CNT sample (Supplementary Fig. 2).

In addition to activity, the ORR selectivity is also evaluated by the electron transfer number and peroxide yield (Supplementary Fig. 3), which were obtained based on the rotating ring-disk electrode (RRDE) method^32^. It can be observed that although the majority of oxygen on Cu/G is reduced to hydroxide via the 4-electron pathway, the amount of peroxide is fairly noticeable at the relatively low potential region. On Cu/CNT-8, however, a remarkable selectivity to hydroxide is presented with the peroxide yield <5%. In comparison, Cu/CNT-4 shows a rather different selectivity, whose peroxide yield is even higher than that of Cu/G in the whole region.

These results clearly demonstrate the importance of carbon substrate nature in designing the SACs. Modulating the carbon substrate from graphene to a proper CNT can tremendously enhance the activity and selectivity to hydroxide of CuN_2_C_2_ SACs towards the ORR, while the CNT substrate with too small or too large diameter worsens the catalytic properties, despite their similar *sp*^2^ hybridized structure. At the same time, Cu/CNT-8 has a high durability in both potentiostatic and potentiodynamic conditions (Supplementary Fig. 4), probably due to its less edge sites than Cu/G and the lower chemical reactivity of substrate than Cu/CNT-4^33,34^.

### Characterization of Cu-SACs

**Fig. 3:**
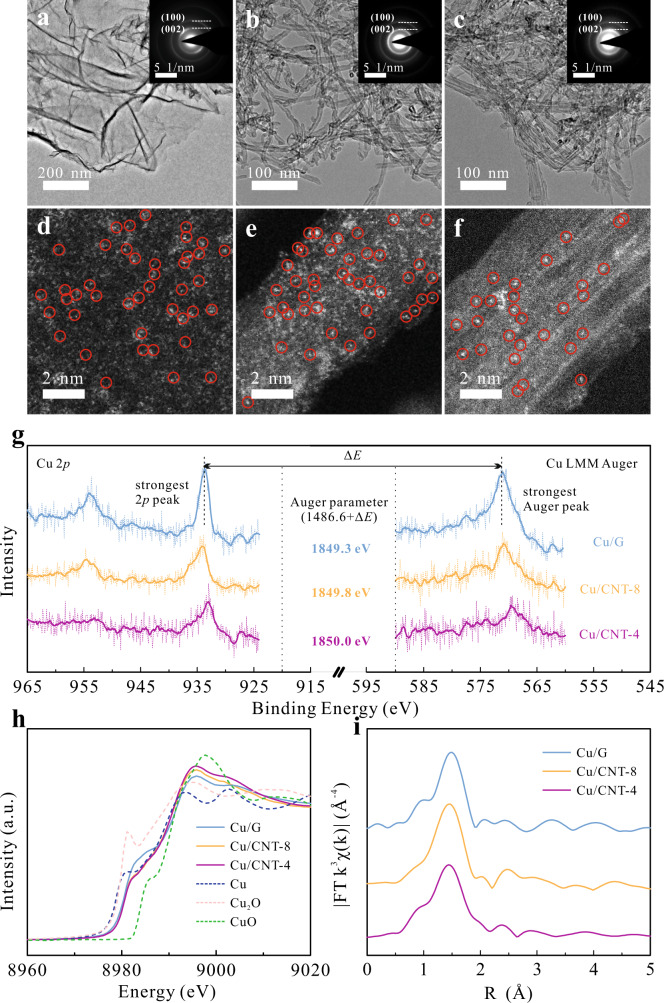
Characterization of Cu-SACs. **a**–**c** TEM images, corresponding SAED patterns (insets in **a**–**c**) and (**d**–**f**) spherical aberration corrected HAADF-STEM images of Cu/G (**a**, **d**), Cu/CNT-8 (**b**, **e**) and Cu/CNT-4 (**c**, **f**), (**g**) Cu 2*p* and Cu LMM Auger XPS spectra of the Cu-SACs, (**h**) Cu *K*-edge XANES spectra of the Cu-SACs with Cu, Cu_2_O, and CuO as references, (**i**) R-space Cu *K*-edge EXAFS of the Cu-SACs.

In order to identify the real ORR active site and reveal the underlying mechanism of carbon substrate nature in tuning the catalytic properties of SACs, the composition and structure of these CuN_2_C_2_ SACs were systematically characterized by experimental characterization and density functional theory (DFT) computation. From the transmission electron-microscopy (TEM) observation (Fig. 3a–c), the Cu/G SAC exhibits a planar sheet-like morphology, while both Cu/CNT samples are the typical cross-stacking nanotube structure. The diameters of Cu/CNT-8 and Cu/CNT-4 SACs measured from the high resolution TEM images (Supplementary Fig. 5) are around 8 and 4 nm, respectively. The bright diffraction rings assigned to graphitic carbon in the selected area electron diffraction (SAED) patterns (insets in Fig. 3a–c) suggest that all the three SACs have the well-defined crystallized *sp*^2^-hybridized carbon nature, without any crystalline Cu-containing species^29^. This observation is verified by their X-ray diffraction (XRD) patterns in Supplementary Fig. 6, where only a strong peak assigned to the C (002) plane can be identified. The atomic dispersion of Cu is evidenced by the sub-nanoscale bright dots observed in the high-angle annular dark-field (HAADF) images from the spherical aberration corrected scanning TEM (AC-STEM) (Fig. 3d–f), and the evenly distributed Cu signals in the energy dispersed X-ray spectroscopy (EDS) results (Supplementary Fig. 7)^35^.

Various elemental analyses were further conducted to gain information on the chemical status of CuN_2_C_2_ SACs. Based on the Cu 2*p* and LMM Auger X-ray photoelectron spectroscopy (XPS) profiles (Fig. 3g), the binding energy difference of Cu 2*p* and Auger peaks, Δ*E*, and its derivative Auger parameter, which is rather sensitive to the oxidation states of Cu, can be calculated^36^. Judging from the similar Auger parameter around 1850 eV, Cu in all the three samples fits common profile of Cu(I)^37^. This conclusion is confirmed by the synchrotron radiation X-ray absorption fine structure (XAFS) analysis. In the Cu *K*-edge X-ray absorption near edge structure (XANES) spectra (Fig. 3h), the absorption edge and white line intensity of all the samples are between the Cu_2_O and CuO references. The enhanced white line intensity for both Cu/CNT SACs, especially Cu/CNT-4, in comparison with Cu/G suggests higher oxidation state of Cu^38^, which should result from the more electron transfer between Cu and the conjugated π bond in graphene than in CNT (Supplementary Table 3).

In the R-space extended XAFS (EXAFS) spectra (Fig. 3i), all the SACs present only one main peak at ca. 1.5 Å, which is distinctive from the Cu and its oxide references (Supplementary Fig. 8) and rules out their presence. The EXAFS spectra can be well fitted using only Cu–C and Cu–N paths (Supplementary Fig. 9), indicating that the Cu atoms on graphene and CNT substrates are stabilized in the form of CuN_2_C_2_ moiety. The fitting parameters summarized in Supplementary Table 4 suggest that the CuN_2_C_2_ moieties on CNT and graphene have the similar coordination structure, providing well guidance for further DFT modeling and analysis.

### DFT computation

With the information of the composition and structure of CuN_2_C_2_ active site, the DFT computation was conducted to look into the detailed electronic and geometric structures of the active site during the operando ORR catalysis. In the modeling process, the graphene moiety and the armchair single-walled CNTs of (59,59) type (diameter ~8 nm) and (29,29) type (diameter ~4 nm) were employed as the full scale substrates, in order to simulate the actual local environment around the CuN_2_C_2_ active site. The three models are shown in Supplementary Fig. 10.

**Fig. 4:**
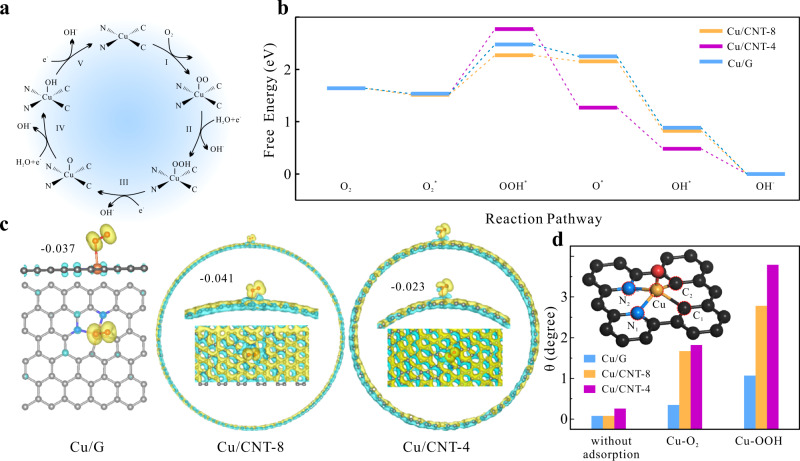
DFT computation results on energy, electron structure, and geometry analysis. **a** Illustration of ORR process on CuN_2_C_2_ active site, (**b**) ORR free energy diagrams on Cu/CNT-8, Cu/CNT-4 and Cu/G, (**c**) side view and top view of the charge density difference for three models with O_2_^*^, where yellow and blue areas represent higher and lower charge density, respectively, and (**d**) geometric descriptor *θ* of CuN_2_C_2_ active site at various stages of ORR for the three models (inset: illustration of the model used for geometry analysis).

The entire ORR process in alkaline media fits the profile of associate pathway with the following five elementary steps according to the DFT calculation (Supplementary Table 5), and illustrated in Fig. 4a^39^. The proposed reaction mechanism is in accordance with most of the reports on the ORR SACs in aqueous systems^2,40,41^.

1 $${}^{\ast }\,+\,{{{{{{\rm{O}}}}}}}_{2}\,\to\, {}^{\ast }{{{{{\rm{O}}}}}}_{2}$$

2 $${}^{\ast }{{{{{\rm{O}}}}}}_{2}\,+\,{{{{{{\rm{H}}}}}}}_{2}{{{{{\rm{O}}}}}}\,+\,{{{{{{\rm{e}}}}}}}^{-}\,\to\, {}^{\ast }{{{{{\rm{O}}}}}}{{{{{\rm{OH}}}}}}\,+\,{{{{{{\rm{OH}}}}}}}^{-}$$

3 $${}^{\ast }{{{{{\rm{O}}}}}}{{{{{\rm{OH}}}}}}\,+{{{{{{\rm{e}}}}}}}^{-}\,\to\, {}^{\ast }{{{{{\rm{O}}}}}}\,+\,{{{{{{\rm{OH}}}}}}}^{-}$$

4 $${}^{\ast }{{{{{\rm{O}}}}}}\,+\,{{{{{{\rm{H}}}}}}}_{2}{{{{{\rm{O}}}}}}\,+\,{{{{{{\rm{e}}}}}}}^{-}\,\to\, {}^{\ast }{{{{{\rm{O}}}}}}{{{{{\rm{H}}}}}}\,+\,{{{{{{\rm{OH}}}}}}}^{-}$$

5 $${}^{\ast }{{{{{\rm{O}}}}}}{{{{{\rm{H}}}}}}\,+\,{{{{{{\rm{e}}}}}}}^{-}{\,\to\, }^{\ast }\,+\,{{{{{{\rm{OH}}}}}}}^{-}$$

where ^*^ represents the active site for the adsorption of intermediates.

The Gibbs free energies of all the related oxygen-containing species adsorbed on the active sites were calculated by the CP2K/Quickstep package, and the free energy diagrams of ORR reaction pathways are presented in Fig. 4b. Based on the calculation results, all the O-containing intermediates involved are preferred to adsorb on the Cu center compared with the neighboring C or N atoms (Supplementary Tables 6, 7), whose geometries are illustrated in Supplementary Fig. 11b, supporting that Cu is the real adsorption and catalytic site for the ORR. Visually, the free energy diagrams of all the three models differ substantially from that of Cu (111) (Supplementary Fig. 11a). The trend of Cu/G shares the similarity with that of Cu/CNT-8, but is distinctive from that of Cu/CNT-4. Nevertheless, the potential determining step (i.e., the most endothermic step) is the same for these models, which is the protonation of ^*^O_2_ to ^*^OOH. The energy difference in the potential determining step for Cu/CNT-8 is the smallest, consistent with its highest ORR activity^42^.

The charge density difference analysis was performed to evaluate the charge transfer between the active site and the O-containing intermediates on the models, and the results are shown in Fig. 4c and Supplementary Fig. 12, where the yellow and blue areas represent the higher and lower electron densities, respectively. For all the models, electron is transferred from Cu onto the adsorbed O-containing intermediates. The Mulliken charge of ^*^O_2_, the reactant of potential determining step, was calculated and given in Fig. 4c. It is found that more negative charge is transferred to ^*^O_2_ on Cu/CNT-8 (−0.041) in comparison with Cu/CNT-4 (−0.023) and Cu/G (−0.037), which results in a more favorable protonation of ^*^O_2_. Sufficient electron transfer promotes the further reduction of adsorbed species and thus the ORR activity, which is well consistent with our experiment results^43^. Since the active sites for the three models are exactly the same in composition, the varied electron transfer capability originates from the substrate structure and its induced operando evolution.

We evaluate the geometry structure evolution of different CuN_2_C_2_ active sites during the reaction process. In order to quantify the geometric structure distortion, a descriptor, *θ*, is introduced, which is calculated by the following equation:

6 $$\theta =\, 360^\circ -\alpha =360^\circ -(\angle {{{{{{\rm{N}}}}}}}_{1}-{{{{{\rm{Cu}}}}}}-{{{{{{\rm{N}}}}}}}_{2}+\angle {{{{{{\rm{N}}}}}}}_{2}-{{{{{\rm{Cu}}}}}}\\ -{{{{{{\rm{C}}}}}}}_{1}+\angle {{{{{{\rm{C}}}}}}}_{1}-{{{{{\rm{Cu}}}}}}-{{{{{{\rm{C}}}}}}}_{2}+\angle {{{{{{\rm{C}}}}}}}_{2}-{{{{{\rm{Cu}}}}}}-{{{{{{\rm{N}}}}}}}_{1})$$

where *α* is the sum of the bond angles between every two neighboring Cu–N or Cu–C bonds, as illustrated in the inset of Fig. 4d. Ideally, for the CuN_2_C_2_ active site embedded in a planar *sp*^2^-hybridized carbon framework, *θ* should be close to 0°. As the coordination asymmetry/substrate curvature varies, or the adsorption species induces, *θ* deviates from 0. In Fig. 4d, *θ* of the pristine CuN_2_C_2_ active site without adsorbing the O-containing intermediates is almost identical for Cu/G and Cu/CNT-8, and slightly increases for Cu/CNT-4, which is induced by its larger curvature with smaller diameter, in accordance with the conclusion on pure CNT^44^. However, when O_2_ is adsorbed on Cu, initiating the ORR, the situation becomes quite different. Cu/CNT-8 undergoes more obvious distortion upon O_2_ adsorption as evidenced by its *θ* variation (Δ*θ*) much greater than that of Cu/G (1.59° vs. 0.26°), even though their original geometry structures are almost identical to each other. Moreover, the structural distortion is more apparent on Cu/CNT-4 with greater *θ* during the whole reaction process.

In addition to the bond angle, the geometry distortion of CuN_2_C_2_ active site is also revealed by the bond length. Taking the Cu–N bond as an example, the average Cu–N bond length elongates after the O_2_ adsorption (Supplementary Table 8). Similar with *θ*, Cu/CNT-4 presents the largest bond length increment, which is sequentially followed by Cu/CNT-8 and Cu/G. These calculation results clearly demonstrate that the three models present distinctive geometry structure distortion during the ORR catalytic process, despite their similar original structures.

### Operando XAFS and mechanism of distortion on ORR

**Fig. 5:**
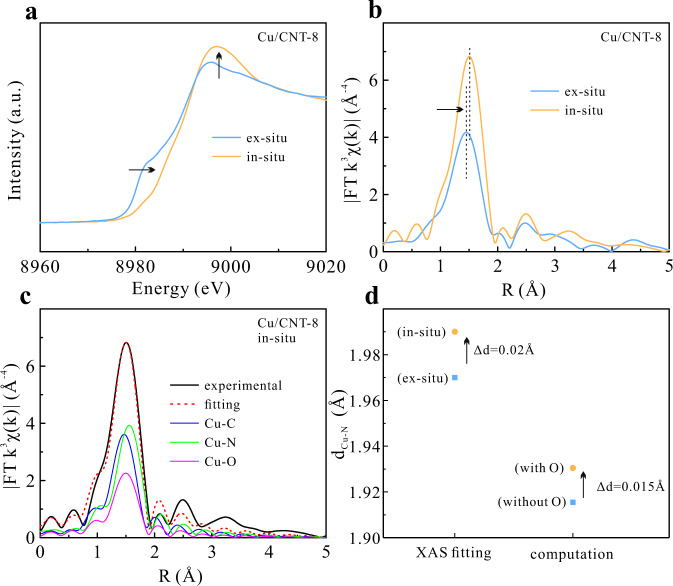
XAFS characterization of Cu/CNT-8. In situ and ex situ Cu *K*-edge (**a**) XANES and (**b**) EXAFS of Cu/CNT-8, (**c**) fitting of in situ R-space EXAFS spectrum of Cu/CNT-8, and (**d**) comparison of Cu–N bond lengths obtained from EXAFS fitting and DFT calculation.

In order to gain the experimental evidence of the geometric structure distortion induced by the O-containing species, we investigate the operando behavior of CuN_2_C_2_ active site via the in situ XAFS on Cu/CNT-8. Comparing with the ex situ condition, the operando Cu *K*-edge XANES (Fig. 5a) clearly shows the positively shifted absorption edge and the enhanced white line intensity, revealing a upshift of Cu oxidation state, which should be ascribed to the thermodynamically favored adsorption of O_2_ on Cu as revealed by the DFT calculation and the subsequent electron transfer driven by the electronegativity^18^. The operando R-space EXAFS spectra (Fig. 5b) shows the increment of magnitude and the positive shift of the main peak, which, based on the fitting results, are attributed to the formation of a new Cu–O coordination with the coordination number of 1 (Fig. 5c and Supplementary Fig. 13). It is noteworthy that from the fitting results, the Cu–N coordination distance of Cu/CNT-8 elongates by 0.015 Å in the operando test, well in accordance with the increment of Cu–N bond upon the O_2_ adsorption from the DFT calculation (Fig. 5d and Supplementary Table 9). The in situ XAFS results provide direct and strong experimental evidence to support Cu is the real active site in the CuN_2_C_2_ moiety for ORR, and the original coordination structure distinctively distorts after the O_2_ adsorption.

The operando geometric structure distortion during the ORR catalysis is directly related to the displacement of metal centers, as a structural response to the adsorption of reaction intermediates. This response depends on the carbon substrate, which is closely correlated to the strain of substrate framework. As the substrate is changed from graphene, the CNT with large diameter, to the CNT with small diameter, curvature increases although they have the same *sp*^2^-hybridized carbon composition. The increase in curvature induces the pyramidalization of *sp*^2^-hybridized C atoms and the misalignment of p orbitals, enhancing the torsional strain in the carbon frameworks^45^. The high strain tends to be released, if possible, through distorting the conjugated framework to a lower energy structure. According to the bond angle analysis (Supplementary Fig. 14 and Supplementary Table 10), the geometry distortion by the newly formed axial Cu–O bond induces the relaxation of neighboring bonds to lower the local curvature, which is thermodynamically favored. This analysis can explain the curvature-dependent distortion levels varying from severe Cu/CNT-4 (the highest strain) to mild Cu/G (the lowest strain).

The adsorption-induced distortion of the geometric coordination environment directly triggers the refinement of the ligand field around the metal Cu atoms, which is a prerequisite for achieving the strong interaction with the adsorbed O-containing species. The structure distortion of Cu/G is the mildest among the three models, and the visible lack of electron density changes between the Cu and O atoms (Fig. 4c) indicates the weak Cu–O hybridization, which is not conducive to the charge transfer from the catalyst to ^*^O_2_. Nevertheless, the too severe distortion in Cu/CNT-4 leads to the over-lengthened Cu–N bond after the ^*^O_2_ adsorption, which impairs the conjugation of Cu 3*d* orbitals with C/N *p*_z_ orbitals, weakening the original Cu–C/Cu–N interaction and thus lowering the electron transfer from N/C atoms to adsorbed ^*^O_2_ via the shared Cu atom. As a result, to achieve the optimal activity of CuN_2_C_2_ SACs, it is vital to modulate the structure distortion to a balanced point, where a strong hybridization of Cu with O can be achieved and meanwhile the original bonds of Cu–N/Cu–C are not excessively undermined. This balance can be reached through rationally tuning the stain of carbon substrates via facilely choosing the different *sp*^2^-hybridized carbon frameworks.

## Discussion

In summary, using the single-atom Cu dispersed on different *sp*^2^-hybridized carbon substrates as the model, we have demonstrated the operando substrate-related geometry distortion of single-atom CuN_2_C_2_ active site and its relationship with the ORR activity. The geometry distortion is revealed to be positively correlated with the torsional strain of carbon substrates reflected by curvature, due to the thermodynamically favored tendency of releasing strain in the curved substrates. Tuning the carbon substrate with a desirable strain is able to reach a delicate balance between achieving the strong Cu–O interaction and maintaining the substantial Cu bonding with the original surrounding atoms. This balance maximizes the charge transferred from Cu atom to O_2_, thus greatly favoring the ORR activity. Our work discloses the structure–function relationship of SACs in terms of carbon substrates, and provides new insights into the mechanism of carbon substrates on the activity expression of embedded single-atom active sites, which can be potentially extended to other catalytic systems. This finding paves a new pathway to further design and the activity promotion of SACs.

## Methods

### Preparation of grapheme oxide (GO)^46^

5 g graphite powder (XFNANO, Co., Ltd.) was added into 180 mL concentrated H_2_SO_4_, and mechanically stirred for 1 h. 90 mL concentrated HNO_3_ and 25 g KMnO_4_ were slowly added into the mixture under ice bath in sequence. The mixture was stirred for 120 h in room temperature. Subsequently, 600 mL ultra-pure water was slowly poured into the mixture and stirred for another 2 h. The slurry turned yellow with bubbling after adding 30 mL H_2_O_2_. The mixture was centrifuged and washed with the mixture of 3 L water, 2 mL concentrated HCl and 5 mL H_2_O_2_, and then with 3 L ultra-pure water. The orange slurry was finally freeze-dried to obtain GO.

### Preparation of oxidized carbon nanotube (OCNT)

CNT (XFNANO, Co., Ltd.) was pretreated in 6 mol L^−1^ HCl solution for 12 h to remove any impurities. After that, the CNT sample was filtrated, washed with ultra-pure water and freeze-dried. 30 mL concentrated sulfuric acid was added into 200 mg treated CNT in a three-neck round-bottom flask. After stirring for 30 min, 10 mL concentrated HNO_3_ was added dropwise, and the mixture was refluxed at 70 °C for 2 h and cooled down to room temperature. OCNT was collected by filtration, washing repeatedly with ultra-pure water and freeze-drying.

### Preparation Cu/G and Cu/CNT catalysts

Typically, 20 mg GO (for Cu–G) or OCNT (for Cu/CNT) and 240 mg dicyandiamide (DICY) were ultrasonically mixed in 20 mL ultra-pure water. The dispersion was stirred overnight and freeze-dried. 100 mg resultant mixture was added into a quartz boat, which was entirely wrapped using a piece of Cu foil. After pyrolyzing at 600 °C for 2 h and 800 °C for another 1 h in Ar, black powder was collected and washed in 0.5 mol L^−1^ O_2_-saturated H_2_SO_4_ for 10 h at 80 °C. The product was washed with ultra-pure water, filtrated, and freeze-dried. After that, the final catalyst was obtained by pyrolyzing at 300 °C for 1 h in Ar. The samples with CNT substrates of 8 and 4 nm diameters were denoted as Cu/CNT-8 and Cu/CNT-4.

### Electrochemical measurement

The mixture of 520 μL water and isopropanol (3:1: in volume), 30 μL 5 wt. % Nafion and 2.6 mg catalyst powder were sonicated to form a homogenous ink. A certain amount of ink was pipetted onto a GC rotating-disk electrode (RDE, 3 mm in diameter) or a rotating ring-disk electrode (RRDE, 5.61 mm in diameter) connected to PINE 636 rotating-disk electrode system, which was adopted as the working electrode. The catalyst loading was 0.4 mg cm^−2^ for RDE and 0.1 mg cm^−2^ for RRDE.

All electrochemical measurements were conducted in a standard three-electrode cell and recorded using CHI 720b electrochemical workstation. Reference and counter electrode were Hg/HgO electrode and Pt foil (1 cm × 1 cm), respectively. All potentials were converted to the reversible hydrogen electrode (RHE) reference scale, and the calibration was conducted by measuring the open circuit potential between Hg/HgO and Pt electrode immersed in H_2_-saturated 0.1 mol L^−1^ KOH at 25 °C.

RDE tests were performed in O_2_ saturated 0.1 mol L^−1^ KOH solution with a scan rate of 10 mV s^−1^ between 1 V and 0 V at different rotating rates, and the background current in Ar saturated KOH solution was subtracted to obtain the ORR polarization curves. RRDE tests were conducted at 1600 rpm and the Pt ring was polarized at 1.2 V. The durability of catalysts was evaluated by both potentiostatic and potentiodynamic procedures. Accelerated durability tests (ADTs) were conducted by potential sweeping between 0.6 V and 1.0 V for 10,000 cycles in O_2_-saturated KOH solution. The negative shift of half-wave potential was obtained based on the ORR polarization curves recorded before and after the ADTs. Potentiostatic *i–t* tests were measured at 1600 rpm at 0.65 V for 10 h, and the current loss was acquired.

Kinetic current density (*i*_k_) was calculated by Koutechy–Levich equation^47^:

7 $$\frac{1}{i}\,=\,\frac{1}{{i}_{{{{{{\rm{k}}}}}}}}\,-\,\frac{1}{B{\omega }^{1/2}}$$

where *i* is the ORR current density, *i*_k_ is the kinetic current density, and *ω* is the rotating rate of RDE.

TOF and MA were calculated by the following equations assuming metal atoms as active sites for ORR^48^:

8 $${{{{{\rm{TOF}}}}}}\,=\,\frac{i{{{{{\rm{k}}}}}}M{{{{{\rm{metal}}}}}}}{{{{{{\mathcal{C}}}}}}{{{{{\rm{cat}}}}}}.\omega {{{{{\rm{metal}}}}}}F}$$

9 $${{{{{\rm{MA}}}}}}\,=\,\frac{i{{{{{\rm{k}}}}}}}{{{{{{\mathcal{C}}}}}}{{{{{\rm{cat}}}}}}.\omega {{{{{\rm{metal}}}}}}}$$

where *i*_k_ is kinetic current density calculated from the K–L equation, *M*_metal_ is molar mass of metal element, *c*_cat._ is mass loading of catalyst on electrode, *ω*_meta*l*_ is mass ratio of metal in catalysts measured by ICP-OES, and *F* is Faraday constant.

Electron transfer number (*n*) and HO_2_^−^ yield (HO_2_^−^%) were calculated by the following equations^49^:

10 $$n\,=\,\frac{4{I}_{{{{{{\rm{d}}}}}}}}{({I}_{{{{{{\rm{d}}}}}}}\,+\,{I}_{{{{{{\rm{r}}}}}}}/N)}$$

11 $${{{{{{\rm{HO}}}}}}}_{2}^{-} \% \,=\,200\,\times\, \frac{{I}_{{{{{{\rm{r}}}}}}}/N}{({I}_{{{{{{\rm{r}}}}}}}/N)\,+\,{I}_{{{{{{\rm{d}}}}}}}}$$

where *I*_d_ is the current recorded on GC disk electrode, *I*_r_ is the current recorded on Pt ring electrode, and *N* is collection efficiency (37%).

### Chaterization

TEM and HAADF scanning transmission electron-microscopy (HAADF-STEM) images were taken on samples dispersed onto Mo grids using a JEOL JEM2010 microscope operated at 200 kV, and a probe aberration corrected JEOL JEM-ARM200CF microscope operated at 200 kV, respectively, for the morphology characterization. Energy dispersive spectroscopy (EDS) was performed on NORAN System 7 equipped with TEM. X-ray photoelectron spectroscopy (XPS) was performed on a Physical Electronics PHI model 5700 instrument using Al *K*α radiation for surface chemistry investigation. PerkinElmer Optima 5300DV ICP-OES System was used to determine the mass fraction of Cu. The crystal structure was analyzed by XRD using a Rigaku D/max-rB diffractometer with Cu *K*α (*λ* = 1.5406 Å) radiation at step size of 0.026°. The Cu *K*-edge (8979 eV) XAFS was collected on a piece of carbon cloth (1 cm × 1 cm) coated with catalyst at a loading of 0.4 mg·cm^−2^ and taped with Kapton film on the back at the 1W1B beamline of Beijing Synchrotron Radiation Facility (BSRF). Ex situ spectra were measured directly in air, and in situ ones were collected by immersing catalyst-coated carbon cloth into O_2_ saturated 0.1 mol L^−1^ KOH solution at 1.0 V in a homemade three-cell system.

### DFT calculations

The zero-gaped armchair CNTs of (29, 29) and (59, 59) were used as substrate to simulate the real diameters of ~4 and ~8 nm in Cu/CNT-8 and Cu/CNT-4, respectively. Periodic boundary conditions were along the tube axis. Cu atoms were fixed in the double-atom vacancies in CNT or graphene structures, with configuration of CuN_2_C_2_, to explore the substrate-dependent electronic and geometric properties. All DFT simulations were carried out using CP2K/Quickstep package^50^. The 2*s*, 2*p* electrons of O, C, and N atoms and the 4*s*, 3*d* electrons of Cu atoms were treated as valence electrons. Perdew–Burke–Ernzerhof functional with Grimme’s dispersion correction was adopted for the treatment of the electron–ion interactions^51^. The core electrons were represented by analytic Goedecker–Teter–Hutter pseudopotentials^52^. The Gaussian basis sets were double-ζ with one set of polarization functions (DZVP)^53^. The plane-wave cutoff for the electron density was 400 Ry.

Cu(111) was modeled by a four layer slab of p(4 × 4) super-cell and the size of simulation box is 10.224 × 10.224 × 21.261. In order to avoid spurious self-interactions, neighboring slabs were separated by a vacuum of 15 Å. Monkhorst-Pack k-point meshes of 4 × 4 × 1 were used for periodic models and an energy cutoff of 400 eV was employed for the plane-wave basis set. The convergence threshold for ionic steps in geometry optimization was 1 × 10^−4^ eV. Geometries were deemed converged when the forces on each atom were below 0.02 eV/Å. A frequency analysis was carried out on the stable states in order to confirm that these represent genuine minima. All of the electronic energies were corrected for zero-point energy (ZPE) contributions.

The change in Gibbs free energy for all intermediates was evaluated using the following relation:

12 $$\varDelta G\,=\,\varDelta E\,-\,T\varDelta S-neU$$

where *E* is the total energy obtained from DFT, *T* is the absolute temperature, *S* is entropy obtained directly from Atkins’ Physical Chemistry^54^, *n* is the number of transferred electrons, and *U* is the operating electrochemical potential vs. standard hydrogen electrode (SHE). In order to calculate the change in *S* of the molecules, O_2_, H_2_, and H_2_O molecules were considered to be in gas phase at room temperature and under ambient pressure, and the S of the adsorbed molecules were negligible^55,56^. The *G* of H_2_O (l), O_2_ (g), and OH^−^ were estimated from the following equations:

13 $${G}_{{{{\mbox{H}}}}_{2}{{\mbox{O(l)}}}}={G}_{{{{\mbox{H}}}}_{2}{{\mbox{O}}}\left({{\mbox{g}}}\right)}+{RT}{ln}\left(\frac{p}{{p}_{0}}\right)$$

14 $${G}_{{{{\mbox{O}}}}_{2}\left({{\mbox{g}}}\right)}=2{G}_{{{{\mbox{H}}}}_{2}{{\mbox{O(l)}}}}-2{G}_{{{{\mbox{H}}}}_{2}{{\mbox{(g)}}}}+4.92$$

15 $${G}_{{{{{{{\rm{OH}}}}}}}^{-}}\,=\,{G}_{{{{{{{\rm{H}}}}}}}_{2}{{{{{\rm{O}}}}}}({{{{{\rm{l}}}}}})}\,-\,{G}_{{{{{{{\rm{H}}}}}}}^{+}}\,=\,{G}_{{{{{{{\rm{H}}}}}}}_{2}{{{{{\rm{O}}}}}}({{{{{\rm{l}}}}}})}\,-\,\frac{1}{2}{G}_{{{{{{{\rm{H}}}}}}}_{2}({{{{{\rm{g}}}}}})}\,+\,{k}_{{{{{{\rm{B}}}}}}}T{{{{{\rm{ln10}}}}}}\,\times\, {{{{{\rm{pH}}}}}}$$

where *R* is the gas constant, *k*_B_ is the Boltzmann constant, *T* = 298.15 K, *p* = 0.035 bar and *p*_0_ = 1 bar. “H^+^ + e^−^” was assumed to be in equilibrium with 1/2 H_2_, at pH = 0 and 0 V potential in SHE.

The adsorption energy (Δ*E*_*ad*_) of the O-containing intermediates was calculated according to the following equation:

16 $$\varDelta {E}_{ad}\,=\,{E}_{{{{{{\rm{total}}}}}}}\,-\,{E}_{{{{{{\rm{substrate}}}}}}}\,-\,{E}_{{{{{{\rm{free}}}}}}}$$

where *E*_total_ is the total energy of the adsorbent and the substrate, *E*_site_ and *E*_free_ are the energy of isolated substrate and free species, respectively.

## Supplementary information

Supplementary Information

## Data Availability

The data that support the findings of this study are available from the corresponding author upon reasonable request.  Source data are provided with this paper.

## Source data

Source Data
