# Supplementary material for: Substrate strain tunes operando geometric distortion and oxygen reduction activity of CuN2C2 single-atom sites
Source: Nat Commun. 2021 Nov 3;12:6335. doi: 10.1038/s41467-021-26747-1 (PMC8566586; doi:10.1038/s41467-021-26747-1)
Supplement: Supplementary file 1 — Supplementary Information [file 41467_2021_26747_MOESM1_ESM.pdf]

Supporting Information inventory

Supplementary Fig. 1 ORR polarization curves of Cu/G and two Cu/CNT samples at different rotating rate, and their corresponding K-L plots

Supplementary Fig. 2 (a) ORR polarization curves and (b) kinetic current density curves for Cu/G and three Cu/CNT samples

Supplementary Fig. 3 Electron transfer number and peroxide yield for Cu/G and Cu/CNT samples

Supplementary Fig. 4 (a) Potentiostatic i-t curves measured at 0.65 V and ADTs curves for (b) Cu/G, (c) Cu/CNT-8, (d) Cu/CNT-4 and (e) Cu/CNT-25

Supplementary Fig. 5 HRTEM images of (a) Cu/G, (b) Cu/CNT-8 and (c) Cu/CNT-4

Supplementary Fig. 6 XRD patterns of Cu/G and two Cu/CNT samples

Supplementary Fig. 7 EDS element mapping of Cu/G and two Cu/CNT samples

Supplementary Fig. 8 R-space Cu K-edge EXAFS spectra of Cu, Cu_2O and CuO

Supplementary Fig. 9 Best-fitted (left) R-space and (right) k-space Cu *K*-edge EXAFS spectra of (a-b) Cu/G and (c-d) Cu/CNT-8

Supplementary Fig. 10 Three models used for DFT calculation

Supplementary Fig. 11 (a) ORR free energy diagram of Cu (111) and (b) illustrations of ORR intermediates

Supplementary Fig. 12 Side view and top view of the charge density difference for (a) Cu/CNT-4, (b) Cu/CNT-8 and (c) Cu/G with OOH^*, O^* and OH^*, where yellow and blue area have higher and lower charge density, respectively,

Supplementary Fig. 13 Best fitting results of in-situ k-space EXAFS of Cu/CNT-8

Supplementary Fig. 14 Illustration of the model for the geometry analysis

Supplementary Table 1 Summary of ORR activity of reported Cu- and other metal-based SACs in 0.1 M KOH

Supplementary Table 2 Cu content and ORR parameters of of Cu/G and two Cu/CNT samples

Supplementary Table 3 Charge analysis results on Cu from three models

Supplementary Table 4 Fitting parameters of best-fitted R-space Cu K-edge EXAFS spectra of Cu/G and Cu/CNT-8

Supplementary Table 5 Free energy change for ORR elementary reactions through different pathway

Supplementary Table 6 Adsorption energy of O-containing intermediates on different site on Cu/G

Supplementary Table 7 Adsorption energy of OOH on different site for three models.

Supplementary Table 8 Average Cu-N length of Cu/CNT-8, Cu/CNT-4 and Cu/G in ORR process obtained from DFT calculation

Supplementary Table 9 Fitting parameters of best-fitted in-situ R-space Cu K-edge EXAFS spectra of Cu/CNT-8

Supplementary Table 10 Geometric descriptor φ of CuN_2 active site for the three models without and with adsorbed O_2

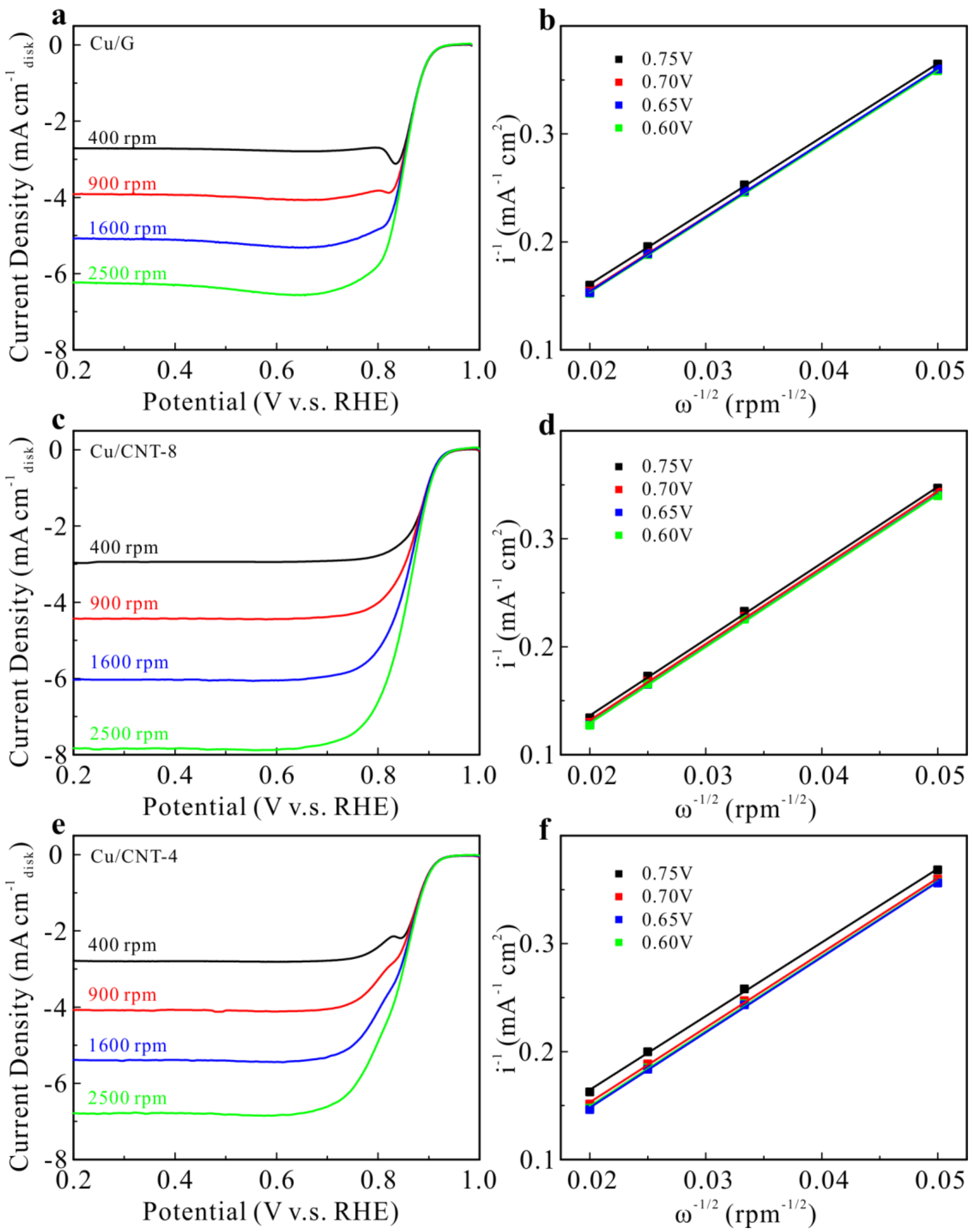

Supplementary Fig. 1 ORR polarization curves of Cu/G and two Cu/CNT samples at different rotating rate, and their corresponding K-L plots

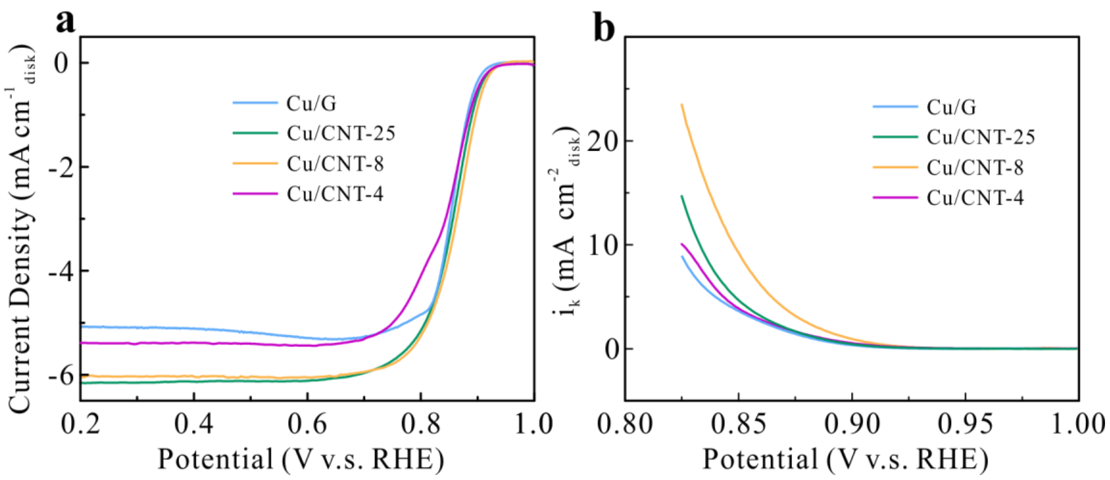

Supplementary Fig. 2 (a) ORR polarization curves and (b) kinetic current density curves for Cu/G and three Cu/CNT samples

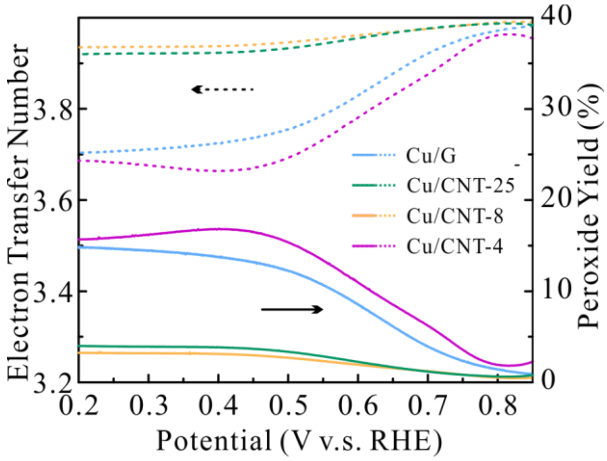

Supplementary Fig. 3 Electron transfer number and peroxide yield for Cu/G and Cu/CNT samples

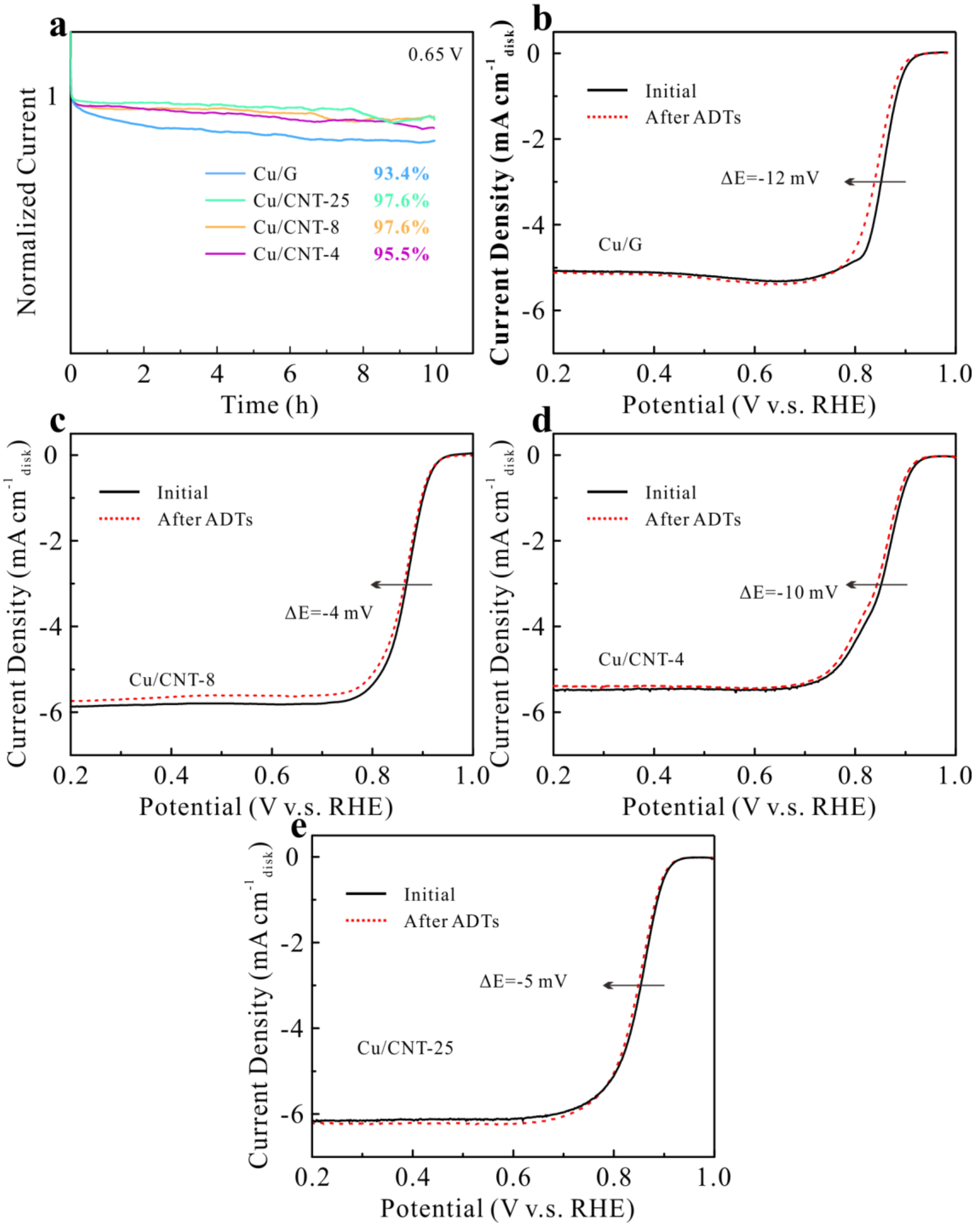

Supplementary Fig. 4 (a) Potentiostatic i-t curves measured at 0.65 V and ADTs curves for (b) Cu/G, (c) Cu/CNT-8, (d) Cu/CNT-4 and (e) Cu/CNT-25

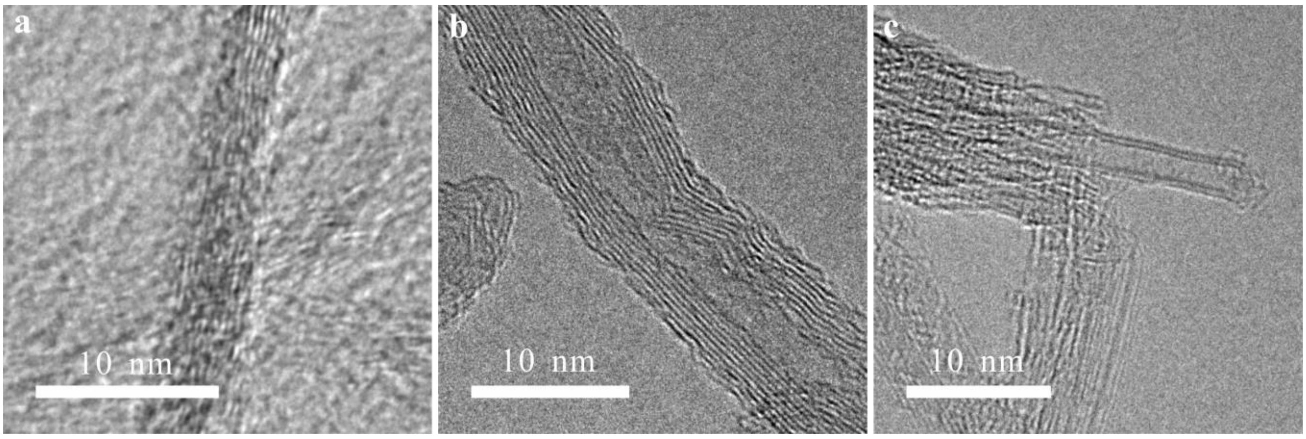

Supplementary Fig. 5 HRTEM images of (a) Cu/G, (b) Cu/CNT-8 and (c) Cu/CNT-4

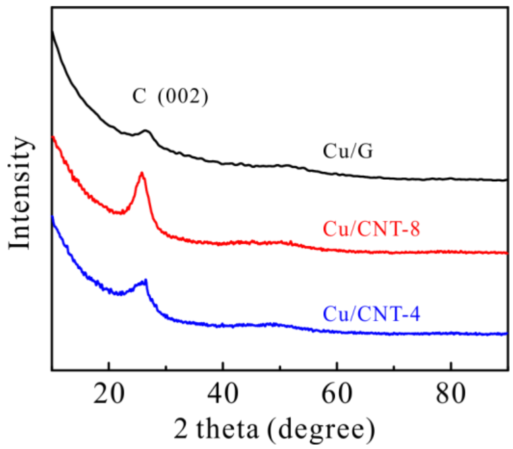

Supplementary Fig. 6 XRD patterns of Cu/G and two Cu/CNT samples

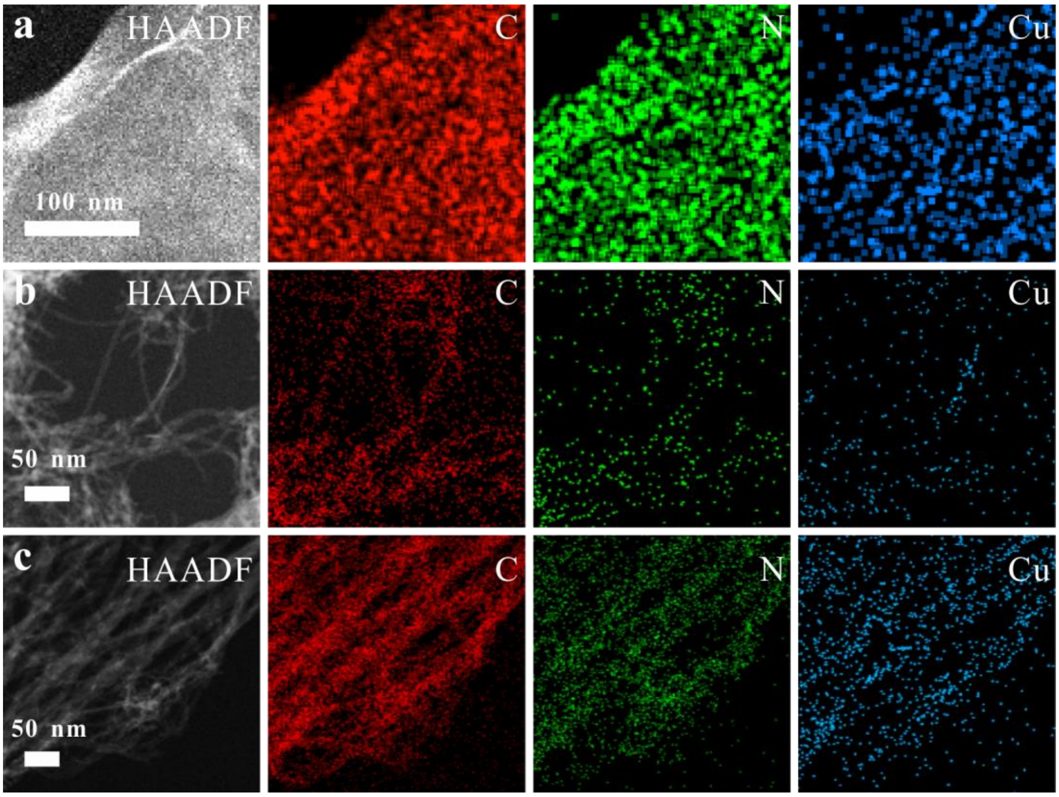

Supplementary Fig. 7 EDS element mapping of Cu/G and two Cu/CNT samples

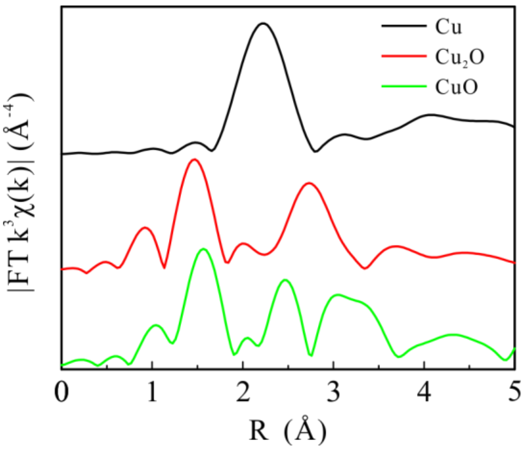

Supplementary Fig. 8 R-space Cu K-edge EXAFS spectra of Cu, Cu_2O and CuO

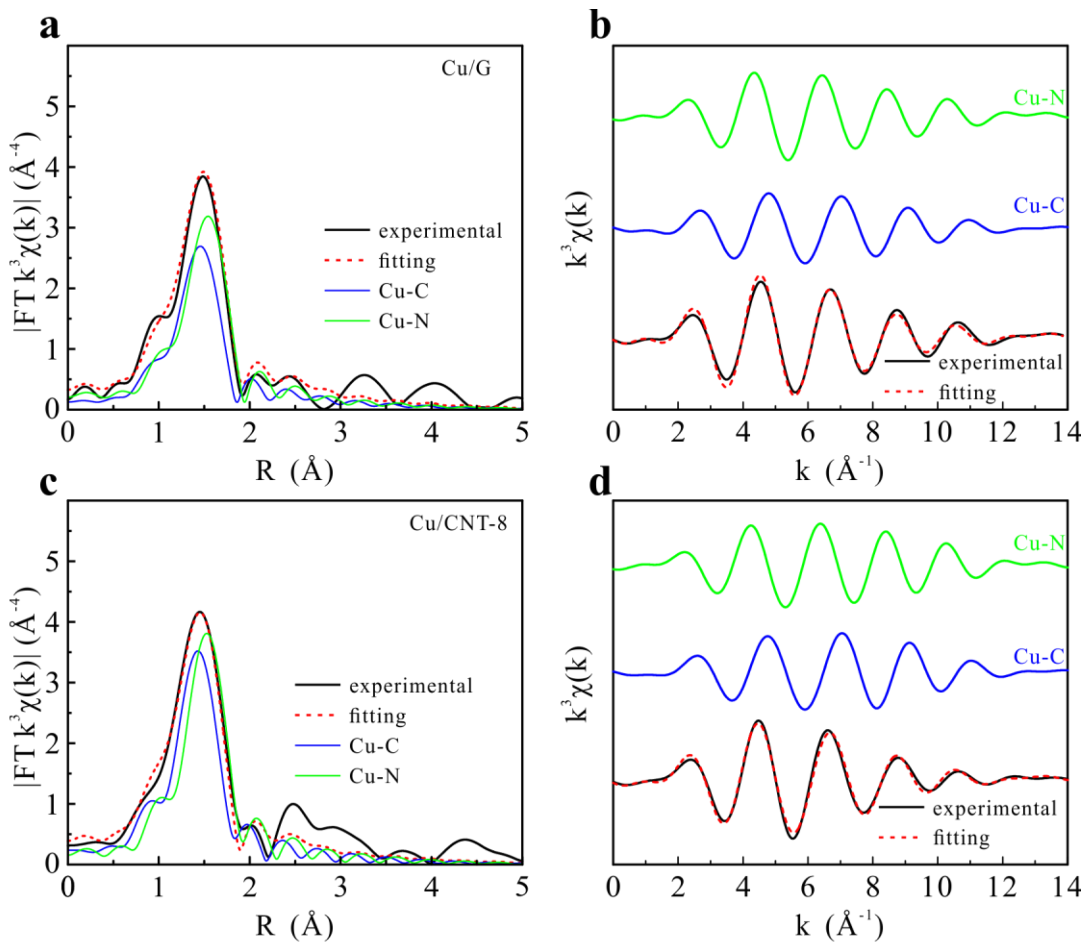

Supplementary Fig. 9 Best-fitted (left) R-space and (right) k-space Cu *K*-edge EXAFS spectra of (a-b) Cu/G and (c-d) Cu/CNT-8

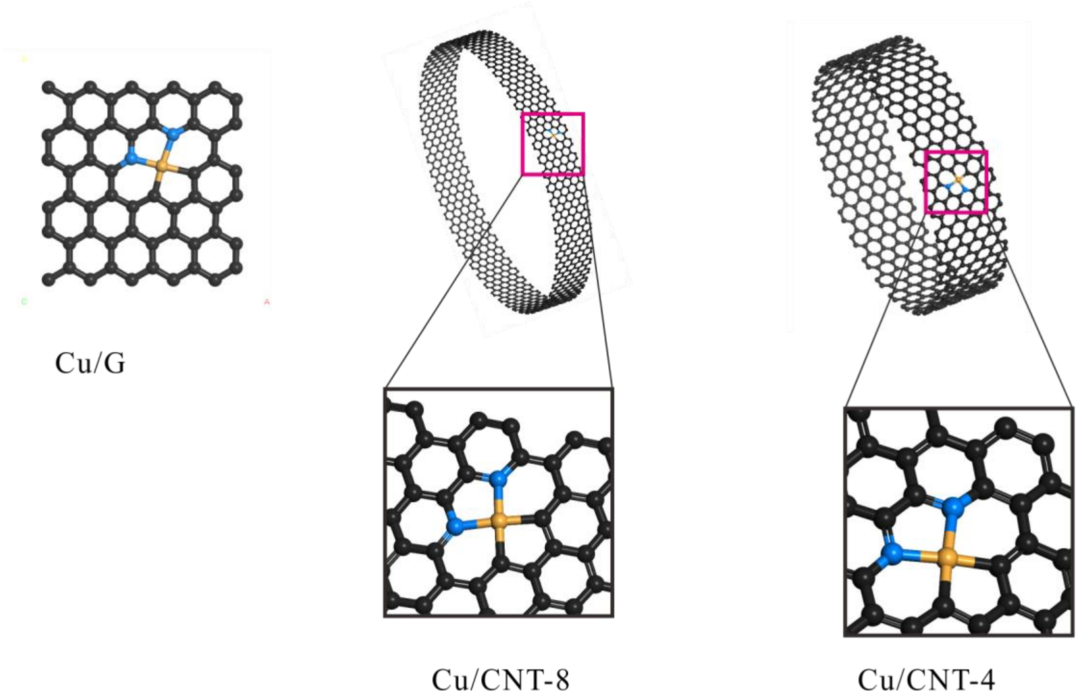

Supplementary Fig. 10 Three models used for DFT calculation

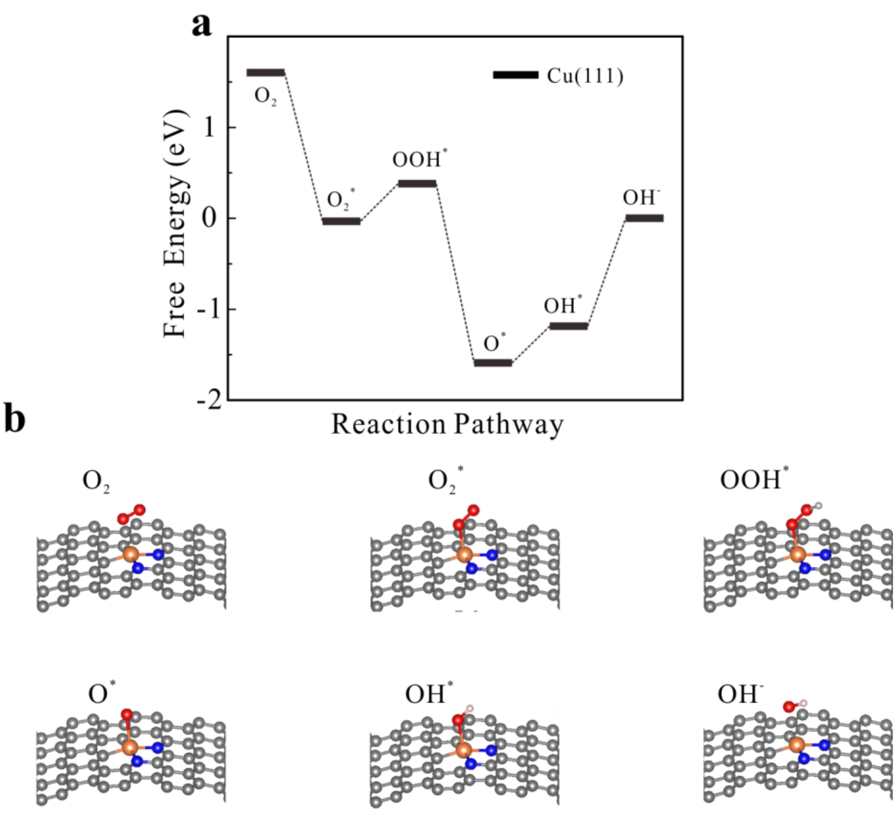

Supplementary Fig. 11 (a) ORR free energy diagram of Cu (111) and (b) illustrations of ORR intermediates

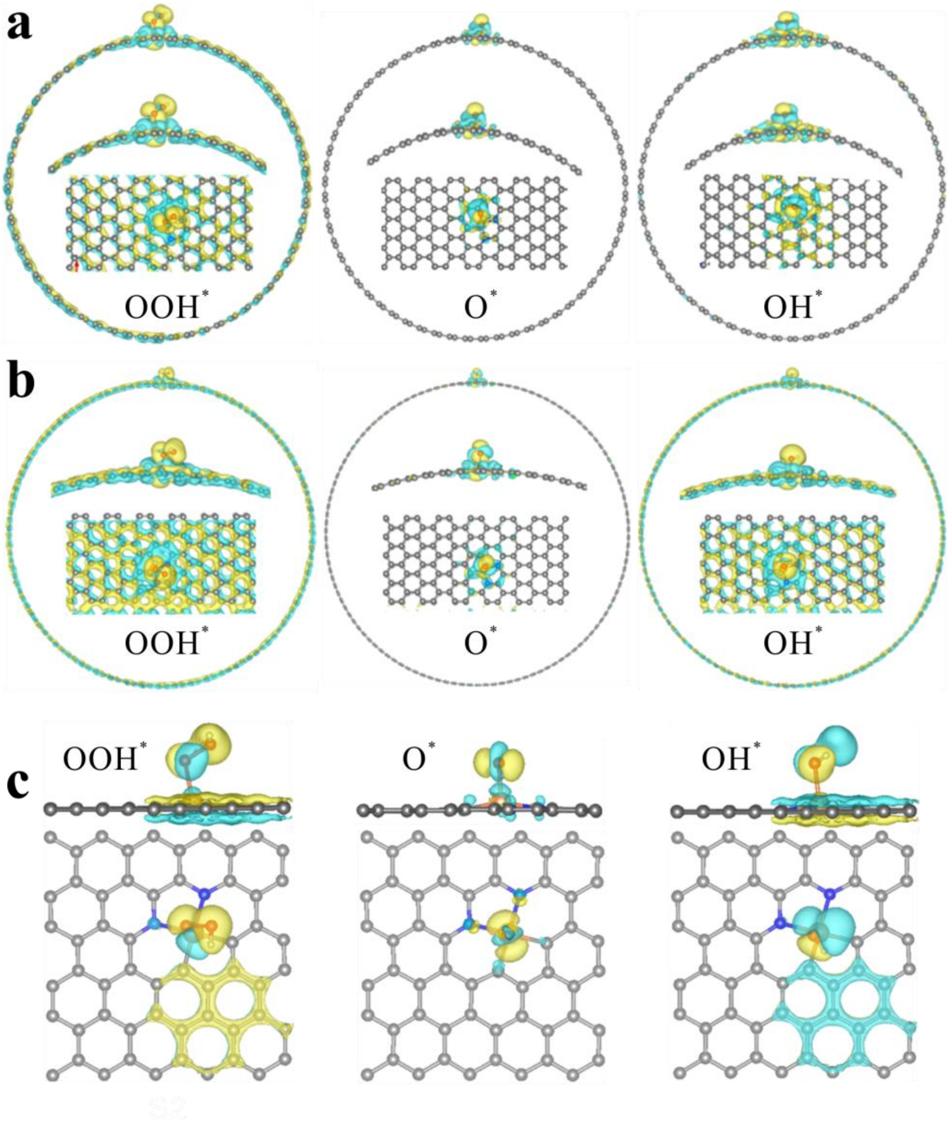

Supplementary Fig. 12 Side view and top view of the charge density difference for (a) Cu/CNT-4, (b) Cu/CNT-8 and (c) Cu/G with OOH^*, O^* and OH^*, where yellow and blue area have higher and lower charge density, respectively,

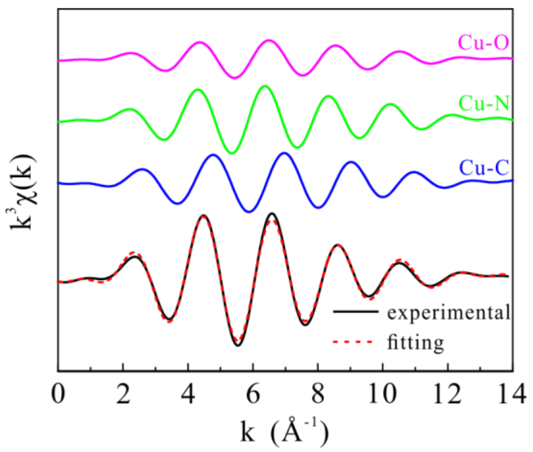

Supplementary Fig. 13 Best fitting results of in-situ k-space EXAFS of Cu/CNT-8

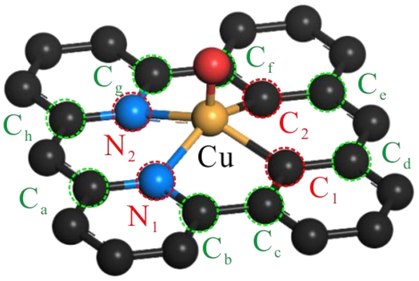

Supplementary Fig. 14 Illustration of the model for the geometry analysis

$$\varphi_1=360°-\beta_1=360°-(\angle C_a\text{-}N_1\text{-}C_b + \angle C_b\text{-}N_1\text{-}Cu + \angle Cu\text{-}N_1\text{-}C_a) \quad (1)$$

$$\varphi_2=360°-\beta_2=360°-(\angle C_c\text{-}C_1\text{-}C_d + \angle C_d\text{-}C_1\text{-}Cu + \angle Cu\text{-}C_1\text{-}C_c) \quad (2)$$

$$\varphi_3=360°-\beta_3=360°-(\angle C_e\text{-}C_2\text{-}C_f + \angle C_f\text{-}C_2\text{-}Cu + \angle Cu\text{-}C_2\text{-}C_e) \quad (3)$$

$$\varphi_4=360°-\beta_4=360°-(\angle C_g\text{-}N_2\text{-}C_h + \angle C_h\text{-}C_2\text{-}Cu + \angle Cu\text{-}C_2\text{-}C_g) \quad (4)$$

$$\varphi=\Sigma\varphi_i/4 \quad (5)$$

where β is the sum of the bond angles between every two neighboring bonds, as illustrated in Supplementary Fig. 14.

Supplementary Table 1 Summary of ORR activity of reported Cu- and other metal-based SACs in 0.1 M KOH

Catalyst	rotating speed / rpm	loading /mg·cm^{-2}	E_{onset} /V	$E_{1/2}$ /V	TOF @0.85 V /e site^{-1} s^{-1}	Ref.
Cu/CNT-8	1600	0.40	0.933	0.863	0.72	This work
Cu-N/C	1600	0.25	0.883	0.804	~0.074	[1]
Cu-N-C	1600	0.50	~0.93	0.869	0.075	[2]
Cu-N©C	2500	0.30	~0.90	-	~0.030	[3]
CPG-900	1000	0.24	~0.93	-	~0.052	[4]
Cu ISs/NC-1000	1600	0.2	0.98	0.855	-	[5]
Cu-N_4-C	1600	0.477	-	0.84	-	[6]
Cu-N-C-ICHP NDs	1600	0.4	0.97	0.85	-	[7]
Co-N_3C_1	1600	0.4	0.904	0.824	0.46 (0.8 V)	[8]
CoSA/N,S-HCS	1600	0.1	0.96	0.85	-	[9]
Ni-N_4/GHSs/ Fe-N_4	1600	0.26	0.93	0.83	-	[10]
Fe_1-HNC-500-850	1600	0.2	~0.9	0.842	-	[11]
Zn/CoN-C	1600	0.25	1	0.861	-	[12]

Supplementary Table 2 Cu content and ORR parameters of of Cu/G and two Cu/CNT samples

	ω_{Cu} /wt.%	E_{onset} /V	$E_{1/2}$ /V	i_k /mA·cm^{-2}		TOF /e site^{-1} s^{-1}		MA /A mg^{-1}	
				0.85 V	0.8 V	0.85 V	0.8 V	0.85 V	0.8 V
Cu/G	5.4	0.917	0.851	3.62	24.16	0.11	0.74	0.17	1.12
Cu/CNT-8	2.13	0.931	0.863	9.24	64.08	0.72	4.99	1.08	7.52
Cu/CNT-4	1.99	0.928	0.844	3.88	14.42	0.32	1.2	0.49	1.81

Supplementary Table 3 Charge analysis results on Cu from three models

	Mulliken Population Analysis	Hirshfeld Charges
Cu/CNT-4	-0.150	-0.674
Cu/CNT-8	-0.167	-0.685
Cu/G	-0.181	-0.700

Supplementary Table 4 Fitting parameters of best-fitted R-space Cu K-edge EXAFS spectra of Cu/G and Cu/CNT-8

Sample	path	N	R /Å	σ^2 /10^{-3}Å^2	ΔE_0 /eV	R-factor
Cu/G	Cu-C	1.8	1.89	5.3	3.8	0.001
	Cu-N	1.9	1.98	5.3	3.8	
Cu/CNT-8	Cu-C	2.0	1.87	3.4	0.2	0.001
	Cu-N	2.0	1.97	3.4	0.2	

Supplementary Table 5 Free energy change for ORR elementary reactions through different pathway

Reactions	ΔG /eV		
	SCNT-40	SCNT-80	G
*+ O_2(g) --- *O_2	-0.10	-0.13	-0.11
O_2(g) + 2* --- 2*O	0.91	2.67	2.85
*OOH + e^- --*O + OH^-	-1.54	-0.16	-0.28
*OOH + H_2O(l)+ e- --- *HOOH + OH^-	-0.62	0.26	0.11

Supplementary Table 6 Adsorption energy of O-containing intermediates on different site on Cu/G

Adsorption site	E_{ad} /eV			
	O_2	OOH	O	OH
Cu	-0.11	-0.35	-2.67	-1.57
C	0.15	-0.2	-2.53	-1.28
N	0.15	-0.14	-2.38	-1.57<br>(move to Cu)

Supplementary Table 7 Adsorption energy of OOH on different site for three models.

Adsorption site	E_{ad} /eV		
	Cu/CNT-4	Cu/CNT-8	Cu/G
Cu	-0.06	-0.56	-0.35
C	0.22	-0.31	-0.20
N	0.19	-0.27	-0.14

Supplementary Table 8 Average Cu-N length of Cu/CNT-8, Cu/CNT-4 and Cu/G in ORR process obtained from DFT calculation

	$d_{Cu\text{-}N}$ /Å (without O_2^*)	$d_{Cu\text{-}N}$ /Å (with O_2^*)	$\Delta d_{Cu\text{-}N}$/Å
Cu/G	1.909	1.922	0.013
Cu/CNT-8	1.915	1.930	0.015
Cu/CNT-4	1.924	1.945	0.020

Supplementary Table 9 Fitting parameters of best-fitted in-situ R-space Cu K-edge EXAFS spectra of Cu/CNT-8

Sample	path	N	R /Å	σ^2 /10^{-3}Å^2	ΔE_0 /eV	R-factor
Cu/CNT-8 (in-situ)	Cu-C	2.0	1.89	3.3	3.2	0.0002
	Cu-N	2.0	1.99	3.3	3.2	
	Cu-O	1.0	1.92	3.3	3.2	

Supplementary Table 10 Geometric descriptor φ of CuN_2 active site for the three models without and with adsorbed O_2

	CuN_2C_2			O_2-CuN_2C_2		
	G	CNT-8	CNT-4	G	CNT-8	CNT-4
φ[1]	0.003	0.058	0.266	0.038	0.015	0.206

[1] illustrate in Supplementary Fig. 14

Supplementary References

1. Lai, Q. *et al.* Directly Anchoring Highly Dispersed Copper Sites on Nitrogen-Doped Carbon for Enhanced Oxygen Reduction Electrocatalysis. *ChemElectroChem* **5**, 1822–1826 (2018).

2. Li, F. *et al.* Boosting oxygen Reduction Catalysis with Abundant Copper Single Atom Active Sites. *Energy Environ. Sci.* **11**, 2263–2269 (2018).

3. Wu, H. *et al.* Highly Doped and Exposed Cu(i)–N Active Sites within Graphene towards Efficient Oxygen Reduction for Zinc–air Batteries. *Energy Environ. Sci.* **9**, 3736–3745 (2016).

4. Wang, J., Wang, K., Wang, F.-B. & Xia, X.-H. Bioinspired Copper Catalyst Effective for both Reduction and Evolution of Oxygen. *Nat. Commun.* **5**, 5285 (2014).

5. Huang, C. *et al.* Copper Isolated Sites on N-Doped Carbon Nanoframes for Efficient Oxygen Reduction. *ACS Sustain. Chem. Eng.* **8**, 14030–14038 (2020).

6. Li, W. *et al.* Bottom-Up Construction of Active Sites in a $Cu–N_4–C$ Catalyst for Highly Efficient Oxygen Reduction Reaction. *ACS Nano* **13**, 3177–3187 (2019).

7. Wang, T. *et al.* Cu,N-Codoped Carbon Nanodisks with Biomimic Stomata-Like Interconnected Hierarchical Porous Topology as Efficient Electrocatalyst for Oxygen Reduction Reaction. *Small* **15**, 1902410 (2019).

8. Hai, X. *et al.* Engineering Local and Global Structures of Single Co Atoms for a Superior Oxygen Reduction Reaction. *ACS Catal.* **10**, 5862–5870 (2020).

9. Zhang, Z. *et al.* Atomically Dispersed Cobalt Trifunctional Electrocatalysts with Tailored Coordination Environment for Flexible Rechargeable Zn–Air Battery and Self-Driven Water Splitting. *Adv. Energy Mater.* **10**, 2002896 (2020).

10. Chen, J. *et al.* Dual Single-Atomic $Ni-N_4$ and $Fe-N_4$ Sites Constructing Janus Hollow Graphene for Selective Oxygen Electrocatalysis. *Adv. Mater.* **32**, 2003134 (2020).

11. Zhang, X. *et al.* A General Method for Transition Metal Single Atoms Anchored on Honeycomb-Like Nitrogen-Doped Carbon Nanosheets. *Adv. Mater.* **32**, 1906905 (2020).

12. Lu, Z. *et al.* An Isolated Zinc–Cobalt Atomic Pair for Highly Active and Durable Oxygen Reduction. *Angew. Chemie - Int. Ed.* **58**, 2622–2626 (2019).
